# Impact of genetic variants on major bleeding after percutaneous coronary intervention based on a prospective multicenter registry

**DOI:** 10.1038/s41598-020-80319-9

**Published:** 2021-01-19

**Authors:** Jung-Joon Cha, Hyung Joon Joo, Jae Hyoung Park, Soon Jun Hong, Tae Hoon Ahn, Byeong-Keuk Kim, WonYong Shin, Sung Gyun Ahn, JungHan Yoon, Yong Hoon Kim, Yun-Hyeong Cho, Woong Chol Kang, Weon Kim, Young-Hyo Lim, HyeonCheol Gwon, WoongGil Choi, Do-Sun Lim

**Affiliations:** 1 Division of Cardiology, Cardiovascular Center, Korea University Anam Hospital, Korea University of College of Medicine, Seoul, South Korea; 2 Division of Cardiology, Severance Cardiovascular Hospital, Yonsei University College of Medicine, Seoul, South Korea; 3 Division of Cardiology, Department of Internal Medicine, Soonchunhyang University Cheonan Hospital, Cheonan, South Korea; 4 Department of Cardiology, Yonsei University Wonju Severance Christian Hospital, Wonju, South Korea; 5 Division of Cardiology, Department of Internal Medicine, Kangwon National University School of Medicine, Chuncheon, South Korea; 6 Department of Internal Medicine, Hanyang University Myongji Hospital, Goyang, South Korea; 7 Department of Cardiology, Gachon University Gil Medical Center, Incheon, South Korea; 8 Department of Internal Medicine, Division of Cardiology, Kyung Hee University Hospital, Kyung Hee University School of Medicine, Seoul, South Korea; 9 Division of Cardiology, Department of Internal Medicine, Hanyang University College of Medicine, Seoul, South Korea; 10 Division of Cardiology, Department of Medicine, Samsung Medical Center, Sungkyunkwan University School of Medicine, Seoul, South Korea; 11 Division of Cardiology, Department of Internal Medicine, Konkuk University College of Medicine, Chungju, South Korea

**Keywords:** Interventional cardiology, Cardiology, Cardiovascular biology, Platelets

## Abstract

Although dual antiplatelet therapy is essential for patients who undergo percutaneous coronary interventions, the risk of bleeding remains an unsolved problem, and there is limited information on the potential relationship between genetic variants and major bleeding. We analyzed the correlations between four major single nucleotide polymorphisms (CYP2C19, ABCB1, PON1, and P2Y12 G52T polymorphisms) and clinical outcomes in 4489 patients from a prospective multicenter registry. The primary endpoint was major bleeding, defined as a Bleeding Academic Research Consortium ≥ 3 bleeding event. The allelic frequencies of ABCB1, PON1, and both individual and combined CYP2C19 variants did not differ significantly between patient groups with and without major bleeding. However, the allelic frequency of the P2Y12 variant differed significantly between the two groups. Focusing on the P2Y12 G52T variant, patients in the TT group had a significantly higher rate of major bleeding (6.4%; adjusted hazard ratio [HR] 2.51; 95% confidence interval [CI] 1.08–5.84; p = 0.033) than patients in the other groups (GG [2.9%] or GT [1.9%]). Therefore, the TT variant of the P2Y12 G52T polymorphism may be an independent predictor of major bleeding.

**Trial registration**: NCT02707445 (https://clinicaltrials.gov/ct2/show/NCT02707445?term=02707445&draw=2&rank=1).

## Introduction

Dual antiplatelet therapy (DAPT) is essential for reducing the occurrence of ischemic events in patients undergoing percutaneous coronary intervention (PCI)^1^. However, the risk of bleeding associated with DAPT remains an unsolved problem, especially in patients with a high bleeding risk (HBR)^2^. The current guidelines recommend short-term DAPT for patients at HBR, although the current evidence is insufficient^3,4^. A recently reported consensus statement has established that customized DAPT should be considered for patients at HBR^5,6^. However, there is a lack of studies on genetic factors that affect bleeding risk.

To address this issue, we evaluated the associations of the single nucleotide polymorphisms of four genes (CYP2C19, ABCB1, PON1, and P2Y12 G52T) with major bleeding; these four genes are known to be involved in the modulation of clopidogrel absorption, metabolic activation, and biologic activity^7–10^. We evaluated these associations in patients who underwent PCI and DAPT over a 1-year follow-up period.

## Results

### Characteristics of the enrolled patients

A total of 4489 patients were enrolled in the current study. The mean loading dose of clopidogrel was 600 mg before the index PCI, and 93.3% of patients continued to receive DAPT for at least 6 months. A total of 122 (2.7%) patients showed major bleeding during the follow-up period. Among the patients showing major bleeding events, 99 patients (81.1%) had non-procedure-related bleeding and 23 patients (18.9%) had periprocedural bleeding. The rates of the individual secondary endpoints of all-cause mortality, cardiac death, non-fatal myocardial infarction, stent thrombosis, target lesion revascularization, and stroke were 1.4%, 0.9%, 0.8%, 0.5%, 5.0%, and 0.6%, respectively. Patients who had major bleeding were more likely to be female; to be older; and to have a medical history of hypertension, diabetes mellitus, hypercholesterolemia, anemia, or chronic kidney disease than patients who did not have major bleeding. The usage of aspirin, clopidogrel, cilostazol, proton-pump inhibitors, calcium-channel blockers, angiotensin-converting enzyme inhibitors, angiotensin II receptor blockers, and beta-blockers was similar between patients with and without major bleeding. However, patients who had major bleeding were less likely to receive statins and short-term DAPT than those who did not (Table 1).

**Table 1:** Patients’ baseline and lesion characteristics and characteristics of in-hospital care.

Characteristics	Total	Patients without major bleeding	Patients with major bleeding	p
	(N = 4489)	(N = 4367)	(N = 122)	
**Baseline characteristics**				
Male sex	3172 (70.7%)	3096 (70.9%)	76 (62.3%)	0.050
Age (years)	64.5 ± 10.7	64.3 ± 10.7	70.0 ± 10.0	< 0.001
Current smoker	1149 (25.6%)	1121 (25.7%)	28 (23.0%)	0.566
Hypertension	2824 (62.9%)	2728 (62.5%)	96 (78.7%)	< 0.001
Diabetes mellitus	1468 (32.7%)	1406 (32.2%)	62 (50.8%)	< 0.001
Hypercholesterolemia	1670 (37.2%)	1640 (37.6%)	30 (24.6%)	0.005
Previous MI	318 (7.1%)	306 (7.0%)	12 (9.8%)	0.307
Previous PCI	630 (14.0%)	608 (13.9%)	22 (18.0%)	0.247
Previous CABG	74 (1.6%)	70 (1.6%)	4 (3.3%)	0.283
Previous CVA	330 (7.4%)	321 (7.4%)	9 (7.4%)	1.000
Congestive heart failure	151 (3.4%)	145 (3.3%)	6 (4.9%)	0.477
Chronic kidney disease	158 (3.5%)	139 (3.2%)	19 (15.6%)	< 0.001
Anemia	1071 (23.9%)	991 (22.7%)	80 (65.6%)	< 0.001
Familial history of CAD	404 (9.0%)	387 (8.9%)	17 (13.9%)	0.077
**Lesion characteristics**				
Multivessel disease	819 (18.2%)	796 (18.2%)	23 (18.9%)	0.954
Presentation with ACS	2381 (53.0%)	2311 (52.9%)	70 (57.4%)	0.378
Left anterior descending artery	2689 (59.9%)	2626 (60.1%)	63 (51.6%)	0.073
Left circumflex artery	1097 (24.4%)	1064 (24.4%)	33 (27.0%)	0.566
Right coronary artery	1488 (33.1%)	1443 (33.0%)	45 (36.9%)	0.429
Left main	184 (4.1%)	178 (4.1%)	6 (4.9%)	0.817
Presence of thrombus	213 (4.7%)	207 (4.7%)	6 (4.9%)	1.000
Thrombosuction	221 (4.9%)	217 (5.0%)	4 (3.3%)	0.523
**Number of stents**				0.187
1	3267 (72.8%)	3183 (72.9%)	84 (68.9%)	
2	970 (21.6%)	941 (21.5%)	29 (23.8%)	
3 or over	252 (5.6%)	243 (5.6%)	9 (7.3%)	
Number of lesions	1.4 ± 1.0	1.4 ± 1.0	1.4 ± 1.0	0.847
Minimal stent size	3.0 ± 0.5	3.0 ± 0.5	2.9 ± 0.4	0.080
Total length of stent	32.1 ± 17.7	32.0 ± 17.6	33.2 ± 20.0	0.509
**In-hospital care**				
**Platelet function test**				
VerifyNow PRU	214.1 ± 76.0	213.7 ± 75.8	229.3 ± 81.8	0.025
**Discharge medication**				
Aspirin	4467 (99.5%)	4347 (99.5%)	120 (98.4%)	0.236
Clopidogrel	4407 (98.2%)	4287 (98.2%)	120 (98.4%)	1.000
Cilostazol	316 (7.0%)	303 (6.9%)	13 (10.7%)	0.160
Proton pump inhibitor	716 (16.0%)	692 (15.8%)	24 (19.7%)	0.311
CCB	1247 (27.8%)	1219 (27.9%)	28 (23.0%)	0.269
Statin	4198 (93.5%)	4091 (93.7%)	107 (87.7%)	0.014
ARB	1533 (34.2%)	1493 (34.2%)	40 (32.8%)	0.822
ACEi	1206 (26.9%)	1166 (26.7%)	40 (32.8%)	0.164
BB	2756 (61.4%)	2688 (61.6%)	68 (55.7%)	0.227
**Duration of DAPT**				
Total duration (days)	322.4 ± 87.4	323.4 ± 86.3	285.8 ± 114.3	< 0.001
> 6 months	4187 (93.3%)	4087 (93.6%)	100 (82.0%)	< 0.001
> 12 months	3645 (81.2%)	3561 (81.5%)	84 (68.9%)	0.001

Data are presented as number (%) or mean (standard deviation).

*ACS* acute coronary syndrome, *MI* myocardial infarction, *PCI* percutaneous coronary intervention, *CABG* coronary artery bypass graft, *CVA* cerebrovascular accident, *CAD* coronary artery disease, *PRU* P2Y12 reaction unit, *DAPT* dual antiplatelet therapy.

### Allelic frequencies according to major bleeding

The allelic frequencies of ABCB1, PON1, and both the individual and combined CYP2C19 variants did not differ significantly between patients with and without major bleeding (Table 2). However, the allelic frequency of the P2Y12 variant differed significantly between patients with and without major bleeding (Table 2).

**Table 2:** Genetic variants of four major single-nucleotide polymorphisms according to the major bleeding.

	Total	Patients without major bleeding	Patients with major bleeding	p
	(N = 4489)	(N = 4367)	(N = 122)	
**CYP2C19**				0.817
Normal metabolizer (*1/*1)	1682 (37.5%)	1633 (37.4%)	49 (40.2%)	
Intermediate metabolizer	2171 (48.4%)	2115 (48.4%)	56 (45.9%)	
*1/*2	1610 (35.9%)	1570 (36.0%)	40 (32.8%)	
*1/*3	561 (12.5%)	545 (12.5%)	16 (13.1%)	
Poor metabolizer	636 (14.2%)	619 (14.2%)	17 (13.9%)	
*2/*2	342 (7.6%)	333 (7.6%)	9 (7.4%)	
*2/*3	245 (5.5%)	238 (5.4%)	7 (5.7%)	
*3/*3	49 (1.1%)	48 (1.1%)	1 (0.8%)	
**PON1**				0.800
RR	539 (12.0%)	522 (12.0%)	17 (13.9%)	
QR	2131 (47.5%)	2074 (47.5%)	57 (46.7%)	
QQ	1819 (40.5%)	1771 (40.6%)	48 (39.3%)	
**ABCB1**				0.597
CC	1824 (40.6%)	1769 (40.5%)	55 (45.1%)	
CT	2060 (45.9%)	2008 (46.0%)	52 (42.6%)	
TT	605 (13.5%)	590 (13.5%)	15 (12.3%)	
**P2Y12 G52T (rs6809699)**				0.025
GG	3407 (75.9%)	3310 (75.8%)	97 (79.5%)	
GT	988 (22.0%)	969 (22.2%)	19 (15.6%)	
TT	94 (2.1%)	88 (2.0%)	6 (4.9%)	

### P2Y12 G52T polymorphisms

The patients were divided into three groups according to the P2Y12 G52T variant observed (GG, GT, and TT). The baseline characteristics of patients in all three groups showed similar trends, including HBR factors such as chronic kidney disease and anemia. The prevalence of the other genetic polymorphisms (CYP2C19, PON1, and ABCB1) did not differ among the three groups (Table 3). The number of treated vessels, the total number of stents, minimal stent size, length of the stent, P2Y12 reaction units, DAPT duration, and discharge medication were also not significantly different among the three groups (Supplementary Table 1).

**Table 3:** Baseline characteristics and genetic variants according to P2Y12 G52T gene polymorphism.

P2Y12 G52T (rs6809699)	GG			GT			TT			p
	(N = 3407)			(N = 988)			(N = 94)			
**Baseline characteristics**										
Male sex	2410 (70.7%)			702 (71.1%)			60 (63.8%)			0.333
Age (years)	64.4 ± 10.7			64.7 ± 10.8			65.6 ± 11.8			0.379
Body mass index (kg/m^2^)	24.6 ± 3.1			24.6 ± 3.0			24.4 ± 2.9			0.703
Current smoker	878 (25.8%)			254 (25.7%)			17 (18.1%)			0.241
Hypertension	2126 (62.4%)			640 (64.8%)			58 (61.7%)			0.384
Diabetes mellitus	1115 (32.7%)			325 (32.9%)			28 (29.8%)			0.827
Hypercholesterolemia	1252 (36.7%)			388 (39.3%)			30 (31.9%)			0.198
Previous MI	236 (6.9%)			73 (7.4%)			9 (9.6%)			0.562
Previous PCI	476 (14.0%)			142 (14.4%)			12 (12.8%)			0.891
Previous CABG	55 (1.6%)			17 (1.7%)			2 (2.1%)			0.910
Previous CVA	246 (7.2%)			75 (7.6%)			9 (9.6%)			0.653
Congestive heart failure	106 (3.1%)			43 (4.4%)			2 (2.1%)			0.130
Chronic kidney disease	127 (3.7%)			25 (2.5%)			6 (6.4%)			0.062
Anemia	812 (23.8%)			238 (24.1%)			21 (22.3%)			0.928
Familial history of CAD	296 (8.7%)			99 (10.0%)			9 (9.6%)			0.428
Presentation with ACS	1823 (53.5%)			509 (51.5%)			49 (52.1%)			0.536
**Genetic variants**										
**CYP2C19**										0.141
Normal metabolizer	1288 (37.8%)			363 (36.7%)			31 (33.0%)			
Intermediate metabolizer	1658 (48.7%)			462 (46.8%)			51 (54.3%)			
Poor metabolizer	461 (13.5%)			163 (16.5%)			12 (12.8%)			
**PON1**										0.705
RR	401 (11.8%)			126 (12.8%)			12 (12.8%)			
QR	1612 (47.3%)			478 (48.4%)			41 (43.6%)			
QQ	1394 (40.9%)			384 (38.9%)			41 (43.6%)			
**ABCB1**										0.930
CC	1378 (40.4%)			410 (41.5%)			36 (38.3%)			
CT	1564 (45.9%)			450 (45.5%)			46 (48.9%)			
TT	465 (13.6%)			128 (13.0%)			12 (12.8%)			

Data are presented as number (%) or mean (standard deviation).

*ACS* acute coronary syndrome, *MI* myocardial infarction, *PCI* percutaneous coronary intervention, *CABG* coronary artery bypass graft, *CVA* cerebrovascular accident, *CAD* coronary artery disease.

**Figure 1:**
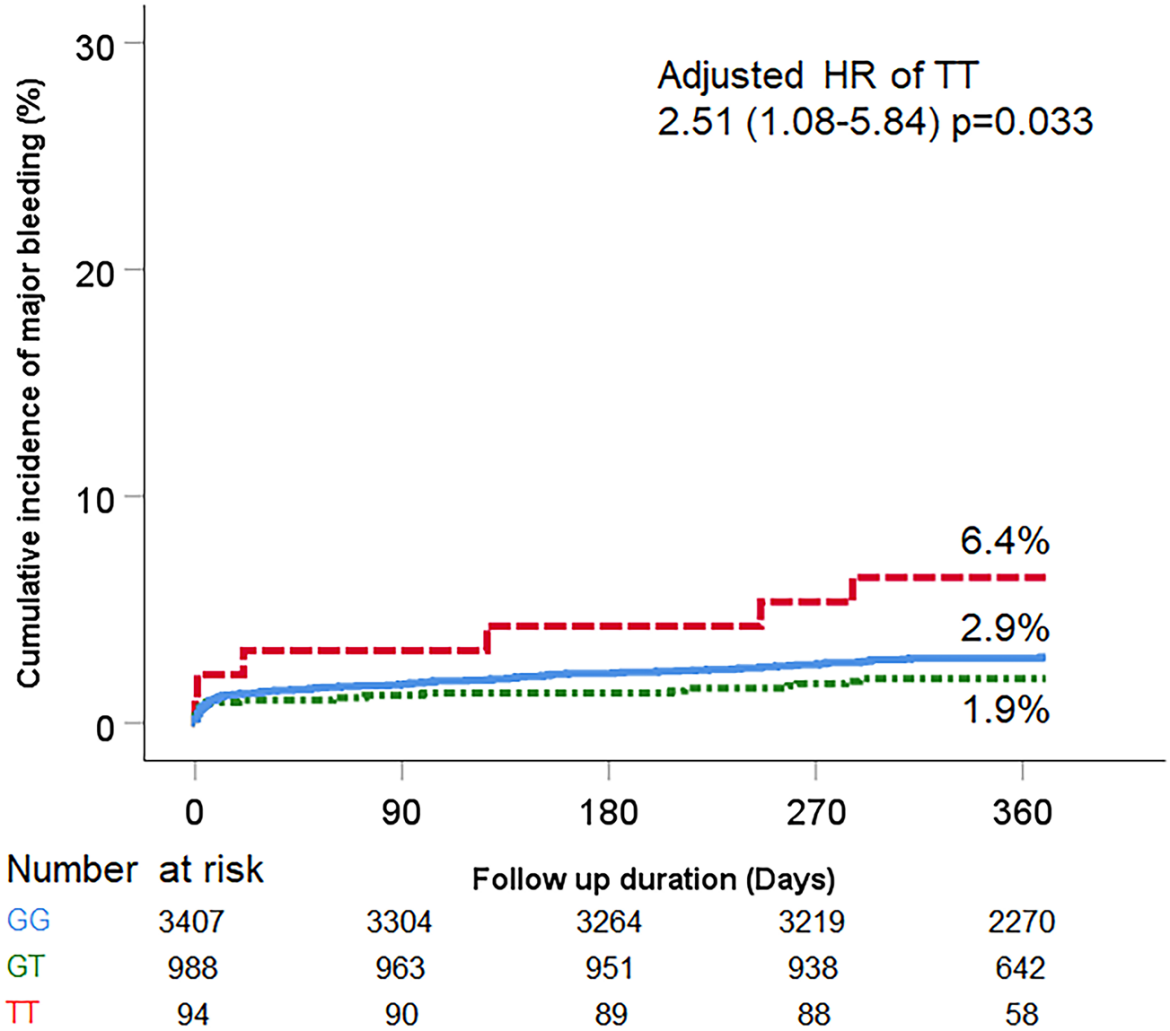
Time-to-event curves through 1 year for major bleeding according to the P2Y12 G52T variant.

Regarding the primary endpoint, the TT group showed the highest incidence of major bleeding compared to the other groups (GG vs. GT vs. TT: 2.9% vs. 1.9% vs. 6.4%, log-rank p = 0.026) (Fig. 1). However, there were no significant differences in the secondary endpoints (any cause mortality, cardiac death, myocardial infarction, stent thrombosis, target lesion revascularization, and stroke) among the groups.

### Multivariate analysis for poor prognostic factors of major bleeding

In the multivariate Cox regression analysis for major bleeding, age (per 1-year increase; adjusted hazard ratio [HR] 1.03; 95% confidence interval [CI] 1.01–1.05; p = 0.004), diabetes mellitus (adjusted HR 1.45; 95% CI 1.00–2.09; p = 0.049), chronic kidney disease (adjusted HR 2.10; 95% CI 1.24–3.55; p = 0.006), anemia (adjusted HR 4.20; 95% CI 2.79–6.34; p < 0.001), and the TT variant (adjusted HR 2.51; 95% CI 1.08–5.84; p = 0.033) were found to be poor prognostic predictors after adjusting for various factors.

## Discussion

We investigated the clinical impact of genetic variants on major bleeding in patients after PCI. The two key findings are as follows: (1) The P2Y12 G52T polymorphism was a predictor of HBR in patients who underwent PCI and received DAPT; and (2) patients with the TT variant had a higher incidence of major bleeding than those with other variants. Furthermore, the TT variant was found to increase the bleeding risk after PCI as per multivariate analysis.

In a recent consensus statement from the Academic Research Consortium, several factors, such as renal failure, liver failure, and anemia, have been suggested as potential contributors to HBR. In patients with HBR, short-term DAPT is recommended^5,6^; however, the current guidelines provide insufficient information regarding the adjustment of the DAPT duration based on genetic information. In a recent report, the genotype-guided DAPT group showed a significant decrease in the incidence of bleeding events without differences in the total combined clinical outcomes compared to the standard-treatment DAPT group^11^. These findings suggest that genotype-guided therapy and precision medicine may improve clinical outcomes in this patient group. In this study, the TT variant of the P2Y12 G52T polymorphism was an independent predictor of major bleeding. Multivariate analysis showed the same tendency as that reported by the current consensus on HBR in terms of renal disease and anemia, suggesting that a novel factor (the TT variant) should be considered to achieve better clinical outcomes in HBR patients.

Previous studies that evaluated the association between genetic variations and clinical outcomes have mainly focused on ischemic outcomes such as cardiac death, myocardial infarction, stent thrombosis, and target lesion revascularization^12^. CYP2C19 is the most commonly studied gene, and several studies on this gene have shown that the gain-of-function group had a higher occurrence of bleeding events than the loss-of-function group^7,13^. However, the prevalence of gain-of-function in Asians with the CYP2C19 variant is very low compared to that in Caucasians with the CYP2C19 variant^8,14,15^. Another study on the Asian population found no association between the gain-of-function allele and bleeding^16^. In addition, ABCB1, PON1, and P2Y12 showed a trend for poor clinical outcomes in terms of ischemic event. However, similar to that for CYP2C19, there was a lack of evidence regarding the association with poor clinical outcomes in terms of major bleeding for these genes^8,9,17,18^.

Our results are similar to those of previous studies that showed that CYP2C19, ABCB1, and PON1 variants were not associated with significant differences in major bleeding. However, our study revealed that the P2Y12 G52T variant had a significant relationship with major bleeding; this is important since studies on the association between P2Y12 and major bleeding have rarely been reported.

Fontana et al. reported that the P2Y12 G52T polymorphism is one of P2Y12 gene polymorphisms^18^. To investigate the correlation between P2Y12 gene polymorphisms and clinical outcomes, i-T744C (rs2046934), C34T (rs6785930), and G52T (rs6809699) were evaluated. Studies on P2Y12 gene polymorphisms have not been widely conducted as compared to studies on CYP2C19 polymorphisms; this may be because the clinical impact of the P2Y12 gene polymorphism is relatively weaker than that of the CYP2C19 polymorphism^8,12,19^. A recent meta-analysis reported clinical outcomes according to P2Y12 gene polymorphisms. Briefly, the study found that P2Y12 gene polymorphisms may be associated with poor clinical outcomes (specifically, ischemic events, such as stent thrombosis and non-fatal myocardial infarction) and have no significant effect on bleeding. However, the meta-analysis evaluated these associations based on studies with relatively small patient populations. In addition, the bleeding analysis only included two polymorphisms (i-T744C and C34T) among the various P2Y12 receptor gene polymorphisms. One study reported that the P2Y12 G52T variant was associated with a higher incidence of major bleeding in patients with ST-elevation myocardial infarction^20^.

A plausible explanation for the lack of association between P2Y12 G52T polymorphisms and major bleeding in previous studies is the low prevalence of the TT variant. In this study, the prevalence of the TT variant of P2Y12 G52T was 2.1%; previous genetic studies have shown a similar prevalence (2–3%)^8,21–24^. Furthermore, the prevalence of the TT variant of P2Y12 G52T was similar across different ethnic populations, unlike that of the gain-of-function allele of CYP2C19^8,25^.

In G52T, the prevalence of the TT variant was relatively lower than that of the other allelic variants (i-T744C and C34T). In context, our findings suggest that previous studies may have underestimated the association between the TT variant and the risk of major bleeding owing to the low prevalence of the P2Y12 G52T variant. Plausible explanations of the mechanism include the following: (1) Inherited defects of the P2Y12 receptor, which has a potential role in platelet function, are related to platelet dysfunction and bleeding diathesis^26^; and (2) there may be differences in ADP-induced maximal aggregation according to the P2Y12 G52T variant^21^. Furthermore, the TT variant was related to higher ADP-induced maximal aggregation than other variants (GG or GT), which may have affected bleeding during the DAPT period. Thus, further studies are needed to address the mechanisms of major bleeding associated with the G52T variant and to confirm our results.

This study has some limitations. First, although a significantly higher occurrence of major bleeding was associated with the TT variant, there may be concerns about applying this result in real-world practice owing to the low prevalence of the TT variant. However, this study is the largest population study to elucidate the correlation between the P2Y12 G52T polymorphism and major bleeding. Second, this study aimed to determine the impact of genetic variants on major bleeding after PCI. Although evaluation of the clinical impact of tailored DAPT was beyond the scope of this study, in the subgroup analysis of patients who received DAPT for 3 months, there was no significant difference in the incidence of major bleeding according to the P2Y12 G52T variant. However, since the suggested duration of DAPT was 1 year in this study, the number of patients who used DAPT for 3 months was relatively small. Thus, we cannot rule out the possibility that the number of patients included in this subgroup analysis was insufficient to achieve adequate statistical power. Third, we evaluated clinical outcomes after a 1-year follow-up after PCI. Although a long-term investigation may provide insights into the clinical impact of P2Y12 G52T polymorphisms, major bleeding is expected to be less frequent after the discontinuation of DAPT after 1 year of PCI.

In conclusion, the TT variant of the P2Y12 G52T polymorphism might be an independent predictor of major bleeding. Therefore, short-term DAPT should be considered for patients with the TT variant to prevent major bleeding.

## Methods

### Study population

**Figure 2:**
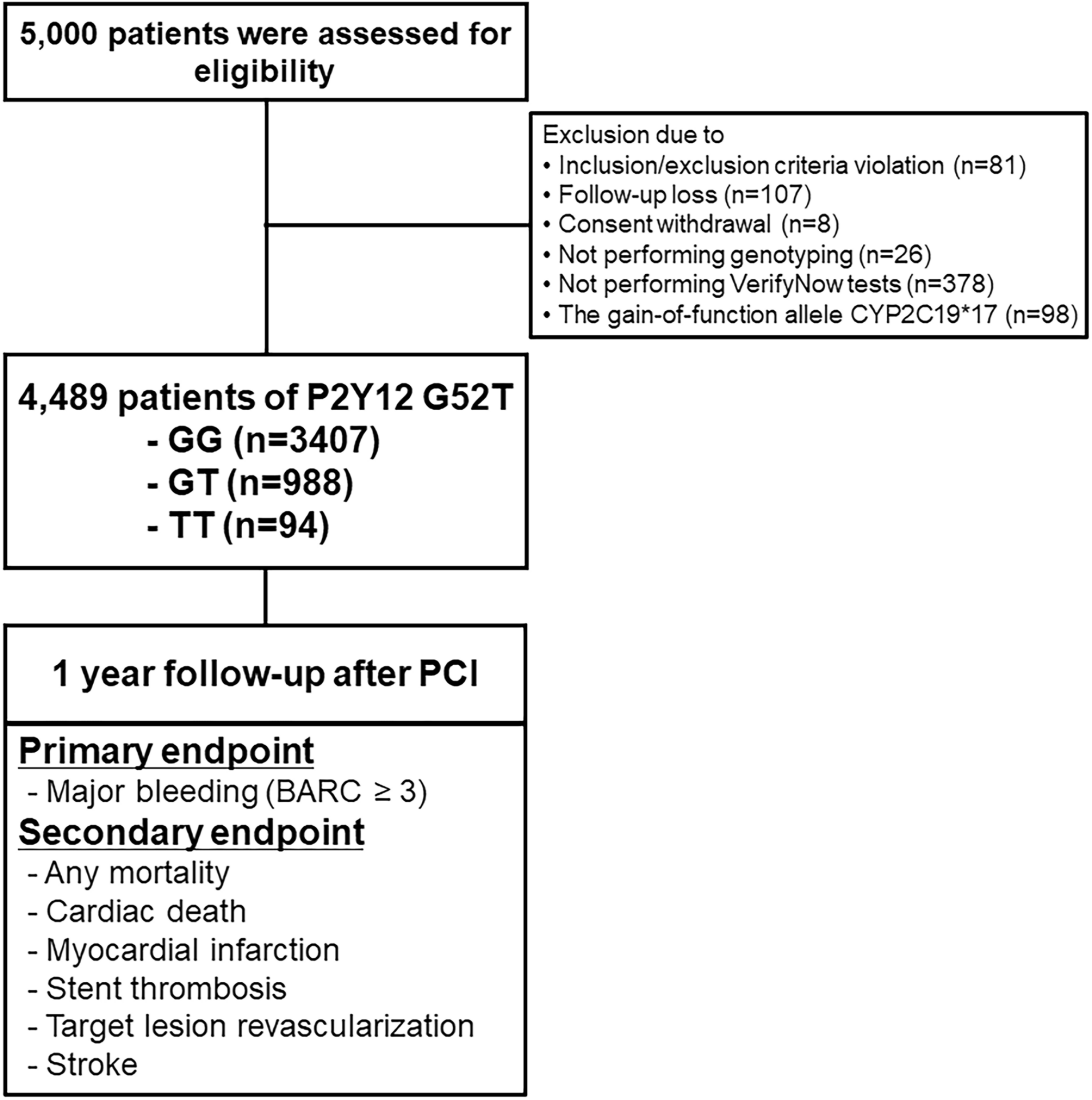
Flow chart.

From 2012 to 2014, the GENIUS study included 5000 patients who underwent PCI for coronary artery disease in 20 tertiary hospitals and investigated the influence of various genotypes on coronary artery stenting outcomes. Among the 5000 patients, 413 patients did not meet inclusion/exclusion criteria, were lost to follow-up, withdrew consent, had missing genotyping results, or had missing platelet function test results. In addition, 98 patients with rapid metabolizers (*17) in CYP2C19 were excluded from the analysis owing to the confounding effects of these substances^16^. Ultimately, 4489 total patients were evaluated in the current study. DAPT was recommended for 1 year (3 months minimum) after the index PCI (Fig. 2). DAPT included aspirin (100 mg daily) and clopidogrel (75 mg daily). No other P2Y12 inhibitors, such as ticagrelor and prasugrel, or anticoagulants, were prescribed after PCI. The study protocol was approved by the Institutional Review Board at each participating center including the Korea University Anam Hospital, Seoul, South Korea. Written informed consent was obtained from all patients at enrollment. This study complied with the Declaration of Helsinki and was registered with ClinicalTrials.gov (number NCT02707445).

### Genotype and platelet reactivity

Measured single nucleotide polymorphisms (SNPs) included CYP2C19*2 (rs4244285), CYP2C19*3 (rs4986893), CYP2C19*17 (rs12248560), ABCB1 (rs1045642), PON1 (rs662), and P2Y12 (rs6809699). The genotype of each SNP was determined by pyrosequencing using a PSQ 96MA Pyrosequencer (Pyrosequencing AB, Uppsala, Sweden), as previously reported^27^. To measure the inhibitory effect of clopidogrel on platelet reactivity, the VeriyfyNow P2Y12 assay (Accumetrics, San Diego, California, USA) was used. Physicians and patients were blinded to residual platelet reactivity and genotype results.

### Endpoint

The primary endpoints were major bleeding, defined as Bleeding Academic Research Consortium (BARC) 3, 4, and 5. The secondary endpoints were any cause mortality, cardiac death, myocardial infarction, stent thrombosis, target lesion revascularization, and stroke.

### Statistical analysis

Comparisons between groups were performed using independent Student’s t-test or analysis of variance (ANOVA) for continuous variables and the chi-square test for categorical variables. Post hoc subgroup analysis was performed based on the baseline characteristics. To estimate the effect of the clinical outcomes, including major bleeding, any cause mortality, cardiac death, myocardial infarction, stent thrombosis, target lesion revascularization, and stroke according to genetic variation, the hazard ratio (HR) was calculated using the Cox proportional hazard model. In the multivariate Cox regression analysis, the HR was adjusted for sex, age, hypertension, diabetes mellitus, previous history of myocardial infarction, previous history of PCIs, congestive heart failure, chronic kidney disease, current smoking status, anemia, clinical presentation to acute coronary syndrome (unstable angina, non-ST elevation myocardial infarction, and ST-elevation myocardial infarction), genetic variants (P2Y12 G52T, CYP2C19, PON1, and ABCB1), duration of DAPT, multivessel involvement, minimal stent size, and total stent length. Two-tailed p-values were used, and p-values of < 0.05 were considered statistically significant. All statistical analyses were performed using the SPSS version 25.0 software (SPSS Inc., Chicago, IL, USA).

## Supplementary Information

Supplementary Information.
