# Supplementary material for: Impact of genetic variants on major bleeding after percutaneous coronary intervention based on a prospective multicenter registry
Source: Sci Rep. 2021 Jan 19;11:1790. doi: 10.1038/s41598-020-80319-9 (PMC7815734; doi:10.1038/s41598-020-80319-9)
Supplement: Supplementary file 1 — Supplementary Information. [file 41598_2020_80319_MOESM1_ESM.docx]

**Impact of genetic variants on major bleeding after percutaneous coronary intervention based on a prospective multicenter registry**

**Short title:** Impact of genetic variants on major bleeding

Jung-Joon Cha^1,†^, Hyung Joon Joo^1,†^, Jae Hyoung Park^1^, Soon Jun Hong^1^, Tae Hoon Ahn^1^, Byeong-Keuk Kim^2^, WonYong Shin^3^, Sung Gyun Ahn^4^, JungHan Yoon^4^, Yong Hoon Kim^5^, Yun-Hyeong Cho^6^, Woong Chol Kang^7^, Weon Kim^8^, Young-Hyo Lim^9^, HyeonCheol Gwon^10^, WoongGil Choi^11^, and Do-Sun Lim^1*^

^1^Division of Cardiology, Cardiovascular Center, Korea University Anam Hospital, Korea University of College of Medicine, Seoul, South Korea

^2^Division of Cardiology, Severance Cardiovascular Hospital, Yonsei University College of Medicine, Seoul, South Korea

^3^Division of Cardiology, Department of Internal Medicine, Soonchunhyang University Cheonan Hospital, Cheonan, South Korea

^4^Department of Cardiology, Yonsei University Wonju Severance Christian Hospital, Wonju, South Korea

^5^Division of Cardiology, Department of Internal Medicine, Kangwon National University School of Medicine, Chuncheon City, South Korea

^6^Department of Internal Medicine, Hanyang University Myongji Hospital, Goyang, South Korea;

^7^Department of Cardiology, Gachon University Gil Medical Center, Incheon, South Korea;

^8^Department of Internal Medicine, Division of Cardiology, Kyung Hee University Hospital, Kyung Hee University School of Medicine, Seoul, South Korea

^9^Division of Cardiology, Department of Internal Medicine, Hanyang University College of Medicine, Seoul, South Korea

^10^Division of Cardiology, Department of Medicine, Samsung Medical Center, Sungkyunkwan University School of Medicine, Seoul, South Korea

^11^Division of Cardiology, Department of Internal Medicine, Konkuk University College of Medicine, Chungju, South Korea

**^*^Corresponding:** e-mail: [dslmd@kumc.or.kr](mailto:dslmd@kumc.or.kr) (D.-S. Lee)

^†^These authors contributed equally to this work.

**Supplement Table 1. Lesion characteristics and characteristics of in-hospital care according to the P2Y12 G52T gene polymorphism.**

| **P2Y12 G52T (rs6809699)** | **GG** | **GT** | **TT** | **p** |
| --- | --- | --- | --- | --- |
|  | **(N=3407)** | **(N=988)** | **(N=94)** |  |
| Lesion characteristics |  |  |  |  |
| Multivessel disease | 618 (18.1%) | 186 (18.8%) | 15 (16.0%) | 0.749 |
| Left anterior descending artery | 2047 (60.1%) | 582 (58.9%) | 60 (63.8%) | 0.589 |
| Left circumflex artery | 833 (24.4%) | 239 (24.2%) | 25 (26.6%) | 0.874 |
| Right coronary artery | 1120 (32.9%) | 344 (34.8%) | 24 (25.5%) | 0.148 |
| Left main | 144 (4.2%) | 39 (3.9%) | 1 (1.1%) | 0.301 |
| Exist of visible thrombus | 156 (4.6%) | 51 (5.2%) | 6 (6.4%) | 0.564 |
| Thrombosuction | 157 (4.6%) | 57 (5.8%) | 7 (7.4%) | 0.173 |
| Number of stents |  |  |  | 0.889 |
| - 1 | 2474 (72.6%) | 725 (73.4%) | 68 (72.3%) |  |
| - 2 | 743 (21.8%) | 209 (21.2%) | 18 (19.1%) |  |
| - 3 or over | 190 (5.6%) | 54 (5.4%) | 8 (8.6%) |  |
| Number of lesions | 1.4 ± 1.0 | 1.4 ± 1.0 | 1.2 ± 0.5 | 0.125 |
| Minimal stent size | 3.0 ± 0.5 | 3.0 ± 0.4 | 2.9 ± 0.5 | 0.196 |
| Total length of stent | 32.0 ± 17.5 | 32.1 ± 18.1 | 33.1 ± 20.0 | 0.833 |
| In-hospital care |  |  |  |  |
| Discharge medication |  |  |  |  |
| Aspirin | 3388 (99.4%) | 985 (99.7%) | 94 (100.0%) | 0.476 |
| Clopidogrel | 3344 (98.2%) | 969 (98.1%) | 94 (100.0%) | 0.405 |
| Cilostazol | 235 (6.9%) | 66 (6.7%) | 15 (16.0%) | 0.003 |
| Proton pump inhibitor | 558 (16.4%) | 140 (14.2%) | 18 (19.1%) | 0.172 |
| CCB | 948 (27.8%) | 270 (27.3%) | 29 (30.9%) | 0.761 |
| Statin | 3181 (93.4%) | 932 (94.3%) | 85 (90.4%) | 0.260 |
| ARB | 1184 (34.8%) | 316 (32.0%) | 33 (35.1%) | 0.266 |
| ACEi | 919 (27.0%) | 265 (26.8%) | 22 (23.4%) | 0.743 |
| BB | 2086 (61.2%) | 611 (61.8%) | 59 (62.8%) | 0.906 |
| Platelet function test |  |  |  |  |
| VerifyNow PRU | 213.6 ± 76.5 | 215.0 ± 75.0 | 224.0 ± 67.6 | 0.392 |
| Duration of DAPT |  |  |  |  |
| Total duration (days) | 322.2 ± 88.2 | 321.2 ± 87.2 | 340.4 ± 54.6 | 0.122 |
| > 6 months | 3173 (93.1%) | 921 (93.2%) | 93 (98.9%) | 0.086 |
| > 12 months | 2769 (81.3%) | 793 (80.3%) | 83 (88.3%) | 0.159 |

Data are number of patients (%) or mean (SD.

CCB, calcium channel blocker; ARB, angiotensin II receptor blocker; ACEi, angiotensin-converting-enzyme inhibitor; BB, beta blocker; PRU, P2Y12 reaction unit; DAPT, dual antiplatelet therapy.
